# The Complex Relationship Between Chronic Obstructive Pulmonary Disease With Cardiovascular Disease and Their Interactions With COVID‐19 Vaccination: A Retrospective Study

**DOI:** 10.1002/iid3.70068

**Published:** 2024-11-18

**Authors:** Muhammad Muneeb Hassan, Sheikh Muhammad Sikandar, Farrukh Jamal, Muhammad Ameeq, Alpha Kargbo

**Affiliations:** ^1^ Department of Statistics The Islamia University of Bahawalpur Bahawalpur Pakistan; ^2^ Department of Statistics DHQ Hospital Muzaffargarh Punjab Pakistan; ^3^ Department of Medicine DHQ Hospital Muzaffargarh Punjab Pakistan; ^4^ Department of Physical and Natural Sciences University of the Gambia Brikama Campus Serrekunda Gambia

**Keywords:** COPD, COVID‐19, CVD, matched cohort, SARS‐CoV‐2

## Abstract

**Background:**

Previously, most researchers explored the association between chronic obstructive pulmonary disease (COPD) and cardiovascular disease (CVD). This study investigates the distinct influence of COVID‐19 vaccination status on patients with both conditions.

**Objective:**

We investigate the relationship between COPD and CVD in a cohort of 838 individuals who presented with both conditions. Our aim is to understand how these conditions interact and how COVID‐19 vaccination status affects patient outcomes.

**Methods:**

A retrospective analysis was conducted on 838 patients with COPD and CVD treated at DHQ Hospital in Muzaffargarh, Punjab, Pakistan, from November 2022 to April 2023. We employed multiple logistic regression and the Wilcoxon signed‐rank test to assess the odds ratio and relative risk of COPD in patients with‐CVD under various conditions. Additionally, we analyzed time‐to‐death and survival using Kaplan–Meier methods.

**Results:**

Findings reveal a 7.95 times higher risk of death in patients without COVID‐19 vaccination compared with those who were vaccinated (95% CI, 6.12–10.33). Conversely, COVID‐19‐vaccinated patients exhibited a 0.221 times lower risk of recovery than their nonvaccinated counterparts (95% CI, 0.08–0.60). We also observed significant differences in time‐to‐death and recovery based on the presence of COPD and CVD, with vaccinated patients generally experiencing milder disease.

**Conclusion:**

Our study assessed the impact of COVID‐19 vaccination status on patient outcomes in patients with overlapping COPD and CVD. Individuals diagnosed with COPD and CVD display significant differences in terms of their probability of survival, with those who have received vaccinations.

## Graphical abstract

Patients for severity without COVID‐19 vaccine had a 7.958 times higher mortality rate than those with it (95% CI, 6.12–10.33). Patients had a lower chance of recovery at 0.22 (95% CI, 0.08–0.60). When hospitalized patients were assessed, those for severity with COVID‐19 vaccine had a similarly higher mortality rate. The estimates of relative risk (RR) with chronic obstructive pulmonary disease (COPD)/without cardiovascular disease (CVD) were at 5.59 (95% CI, 4.68–6.67), and the estimates of RR without‐COPD/with‐CVD observed at 3.79 (95% CI, 3.20–4.48), which denoted greater than the estimates of RR in with‐COPD/without‐CVD patients. The Wilcoxon signed‐rank test clearly highlighted that the null hypothesis is totally rejected at *p* < 0.05, the median of differences between with or without COPD and CVD.

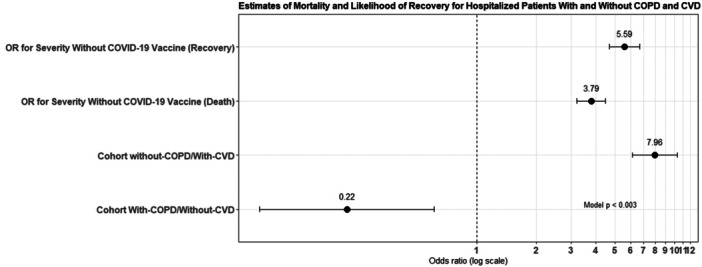

**Abbreviations**
COPD: chronic obstructive pulmonary disease
CVD: cardiovascular disease
DM: diabetes mellitus
HTN: hypertension
LAMA: left against medical advice
LRTI: lower respiratory tract infection
PHC: Punjab Health Council
SARS‐CoV‐2: Severe Acute Respiratory Syndrome Corona Virus‐2
WHO: World Health Organization

## 1 Introduction

Chronic obstructive pulmonary disease (COPD) is the third leading cause of death, accounting for 3.2 million deaths around the world [1]. The global prevalence of COPD is estimated to be more than 250 million, and is expected to rise further in accordance with rising smoking rates and air pollution [2, 3]. Shortness of breath, coughing, wheezing, and fatigue are just some of the COPD symptoms that can drastically decrease the quality of life and contribute to the disease's high mortality rate. Long‐term exposure to poisonous substances (such as air pollution and workplace dust), particularly tobacco smoke, commonly causes COPD [2].

Cardiovascular disease (CVD) is predicted to be responsible for approximately 17.9 million deaths on the worldwide level. This accounts for approximately one‐third of all deaths worldwide [4, 5]. CVD also has a high global prevalence, with an estimated 523 million cases [6]. Prevention and early intervention are essential for lowering CVD rates because the major risk factors, such as lack of physical activity, poor diet, smoking, and obesity, are all modifiable [7]. CVD outcomes have improved as a result of advances in medical treatment and technology, but it remains a significant public health problem requiring ongoing attention and investment [8].

COPD and CVD are recognized as prominent contributors worldwide to morbidity and mortality. In recent years, the COVID‐19 pandemic has added another layer of complexity to the relationship between COPD, CVD, and other chronic diseases [9, 10]. The Severe Acute Respiratory Syndrome Corona Virus‐2 (SARS‐CoV‐2) causes a respiratory illness that primarily affects the lungs but can also directly impact the heart and blood vessels. The most significant risk factor for both diseases (COPD and CVD) is smoking, which damages both the lungs and the cardiovascular system. Age, genetics, air pollution, inactivity, obesity, diabetes, high blood pressure, high cholesterol, and family history are other potential risk factors associated with both CVD and COPD [11].

In comparison to the overall population, individuals diagnosed with CVD exhibit a greater prevalence of COPD. The relationship between the two diseases is bidirectional, and the mechanisms underlying this association are complex and multifactorial, with evidence indicating that COPD can directly impact the development and progression of CVD. Although the exact mechanisms linking them are still being investigated, there are a few different ways in which this condition can indirectly cause CVD problems [12]. A study found that COVID‐19 vaccines significantly reduced hospitalization rates and severe outcomes in COPD patients, emphasizing the importance of vaccination in this vulnerable group. Another study demonstrated that COVID‐19 vaccination led to lower infection rates and reduced mortality in individuals with CVD, reinforcing the protective benefits of COVID‐19 vaccination in patients with‐CVD [13, 14].

The COVID‐19 pandemic caused by SARS‐CoV‐2 has had a significant impact on healthcare systems worldwide, including Pakistan. Respiratory symptoms from the virus increase morbidity and mortality in COPD patients. Pre‐existing CVD and COPD increase COVID‐19 risk of severe illness and death, according to recent reports. Pakistan's leading cause of death, CVD, is also common [15, 16]. The ratio of affected patients in Pakistan is constantly changing due to the dynamic nature of the pandemic. Pakistan reported over 3 million COVID‐19 cases and 80,000 deaths as of February 2023. Pakistan has a 6% COPD and 22% CVD prevalence [17, 18]. This may involve a multidisciplinary approach with respiratory and cardiovascular specialists to optimize outcomes and health. Healthcare providers in Pakistan have identified and managed risk factors to combat COVID‐19 [19, 20]. The implementation of vaccination protocols and infection control measures has the potential to mitigate the possibility of COVID‐19‐associated complications in patients with‐CVD and COPD within the context of the ongoing pandemic in Pakistan.

This study may also provide insights into potential targets for future therapeutic interventions aimed at reducing the burden of COPD, CVD, and COVID‐19. The main reason for the existence of this study was to comprehensively examine the relationship between COPD and CVD in patients, with a particular focus on the associated outcomes. Practical insights for healthcare professionals can be gained by examining the potential effects on patient management strategies, especially in dealing with the complex interplay between COPD, CVD, and COVID‐19 vaccination status. We aim to contribute to the ongoing discussion about optimal patient care and public health initiatives by considering the practical implications of our research in the context of these interconnected health conditions.

## 2 Materials and Methods

The hospital (serving both rural and urban regions throughout four subdistricts) conducted COPD and CVD testing on 838 male and female patients [21]. The hospital statistical officer reports that the following patients' records will be maintained through the year 2021–2022; the total number of OPD (outdoor patients/outpatient department), indoor, COPD with CVD, and COPD (vaccinated and nonvaccinated patients with COVID–19) were 22731, 9342, 1047, and 1029, respectively. COPD was diagnosed using the postbronchodilator FEV1/FVC < 0.7 of the predicted value, in accordance with the diagnostic practices in our study. COPD patients with a history of more than 20 pack years of smoking were included. Patients with both CVD and COPD were included in this study. The diagnosis of CVD and COPD was confirmed by the medical records. Cases of COVID‐19 (vaccinated/nonvaccinated) reported between January 2021 and December 2022 were included in the study. The verification of COVID‐19 cases was conducted using real‐time reverse transcription polymerase chain reaction in laboratories that have received accreditation from the Punjab Health Council and the Research Institute of Nishtar Medical College and University in Multan. The adoption of spirometry, a standard procedure for assessing respiratory function, was employed to detect instances of COPD. The data and information were gathered by a healthcare practitioner, such as a nurse or physician, subsequent to a standard physical examination. The interview form was filled out by hand and collected from the patients. The data collection for this study was conducted in February 2022, and the study covered from November 2022 to April 2023, employing the simple random sample technique. The study employed Kaplan–Meier curves to depict mortality and survival rates stratified by COPD and CVD status. The Wilcoxon rank‐sum test is employed to partition patients with‐CVD and COPD into two distinct groups, facilitating the comparison of their respective survival rates. The purpose of multiple linear regressions in statistics is to predict the value of a response variable using a number of potential explanatory variables.

Smoking = *β*_0_ + *β*_1_(diabetes mellitus) + *β*_2_(hypertension) + *β*_3_(obesity) + *β*_4_(inhaler duration) + *β*_5_(dyslipidemia) + *β*_6_(community‐acquired pneumonia) + *β*_7_(smoking history) + *β*_8_(severity with COVID‐19 vaccination) + *β*_9_(severity without COVID‐19 vaccination).

By the help of multiple regression, smoking with respect to time taken as an dependent variable and the disease which affect the patient like diabetes mellitus (DM), hypertension (HTN), obesity, inhaler duration, dyslipidemia, community‐acquired pneumonia, severity with vaccination, and severity without vaccination, which are all independent variables. All statistical analysis was done in SPSS‐22, MathType, and R.

### 2.1 Hypothesis

Diabetes mellitus, hypertension, obesity, longer duration of inhaler, dyslipidemia, community‐acquired pneumonia, smoking history, COVID‐19 vaccine, and without COVID‐19 vaccine will be more prevalent in those with COPD and CVD, in comparison to those without these conditions.

## 3 Results

There were a total of 838 COPD patients who were hospitalized with CVD, 745 of which were male and 93 of them female. The patients' ages ranged from 31 to more than 71, and all of them suffered from COPD and CVD. To form an appropriate group, smoking histories ranging from more than 20 years were collected from participants. The patient's death and survival rate ratio is calculated using independent variables such as diabetes, HTN, obesity, inhaler duration, dyslipidemia, and community‐acquired pneumonia, as well as severity with and without COVID‐19 vaccination. At *p* < 0.05, all variables are significant, as shown in Table 1.

**Table 1:** Descriptives of COPD patients with‐CVD.

Characteristics	Value (%) *N* = 838	Mean (SD)	95% Confidence interval for mean		*p* value*
			Lower	Upper	
Gender	—	0.11 (0.314)	0.093–0.130		
Male	745 (88.90)	—	—		0.001
Female	93 (11.09)	—	—		
Age	—	1.28 (0.449)	1.250–1.310		
31–51	604 (72.07)	—	—		0.001
51–71 and above	234 (27.92)	—	—		
Area**	—	0.52 (0.500)	1.830–1.980		0.001
Rural	185 (22.02)	—	—		
Urban	653 (77.92)	—	—		
Subdistrict A	452 (53.93)				
Subdistrict B	105 (13.52)				
Subdistrict D	191 (22.79)				
Subdistrict E	90 (11.69)				
Smoking history (pack year)	—	2.71 (1.187)	2.610–2.890		0.001
	—	—	—		
20–25	314 (37.47)	—	—		
26–30	183 (21.83)	—	—		
30 and above	341 (40.69)	—	—		
Socioeconomic status	—	0.78 (0.415)	0.722–0.820		
Patients with middle income	185 (22.01)	—	—		
Patients with poor income	653 (78.02)	—	—		0.001
Duration of inhaler (month)	—	3.19 (1.043)	3.120–3.260		0.001
1–5	20 (2.32)	—	—		
6–10	221 (26.37)	—	—		
11–15	291 (34.72)	—	—		
16–20	195 (23.26)	—	—		
Greater than 21	111 (13.24)	—	—		
Diabetes mellitus	218 (26.01)	0.27 (0.439)	0.230–0.290		0.001
Hypertension	583 (69.57)	0.68 (0.468)	0.670–0.730		0.001
Obesity	184 (21.95)	0.23 (0.425)	0.180–0.260		0.001
Dyslipidemia	380 (45.34)	0.46 (0.488)	0.410–0.480		0.001
Community‐acquired pneumonia	120 (14.31)	0.15 (0.350)	0.119–0.169		0.001
Severity with COVID‐19 vaccination	150 (17.89)	0.88 (0.327)	0.860–0.900		0.001
Severity without COVID‐19 vaccination	688 (82.10)	0.19 (0.384)	0.150–0.210		0.001
With‐COPD/without‐CVD	379 (45.22)	0.45 (0.498)	0.420–0.490		0.001
Without‐COPD/with‐CVD	459 (54.77)	0.67 (0.472)	0.640–0.700		0.001
Event (death)	100 (11.93)	0.03 (0.181)	0.020–0.050		0.001

Abbreviations: COPD, chronic obstructive pulmonary disease; CVD, cardiovascular disease.

* *p* value (< 0.05) is significant.

** Area covered for the patient of COPD patients with‐CVD namely Subdistrict A (Muzaffargarh), Subdistrict B (Kot Addu), Subdistrict C (Ali pur), and Subdistrict D (Jatoi).

**Figure 1:**
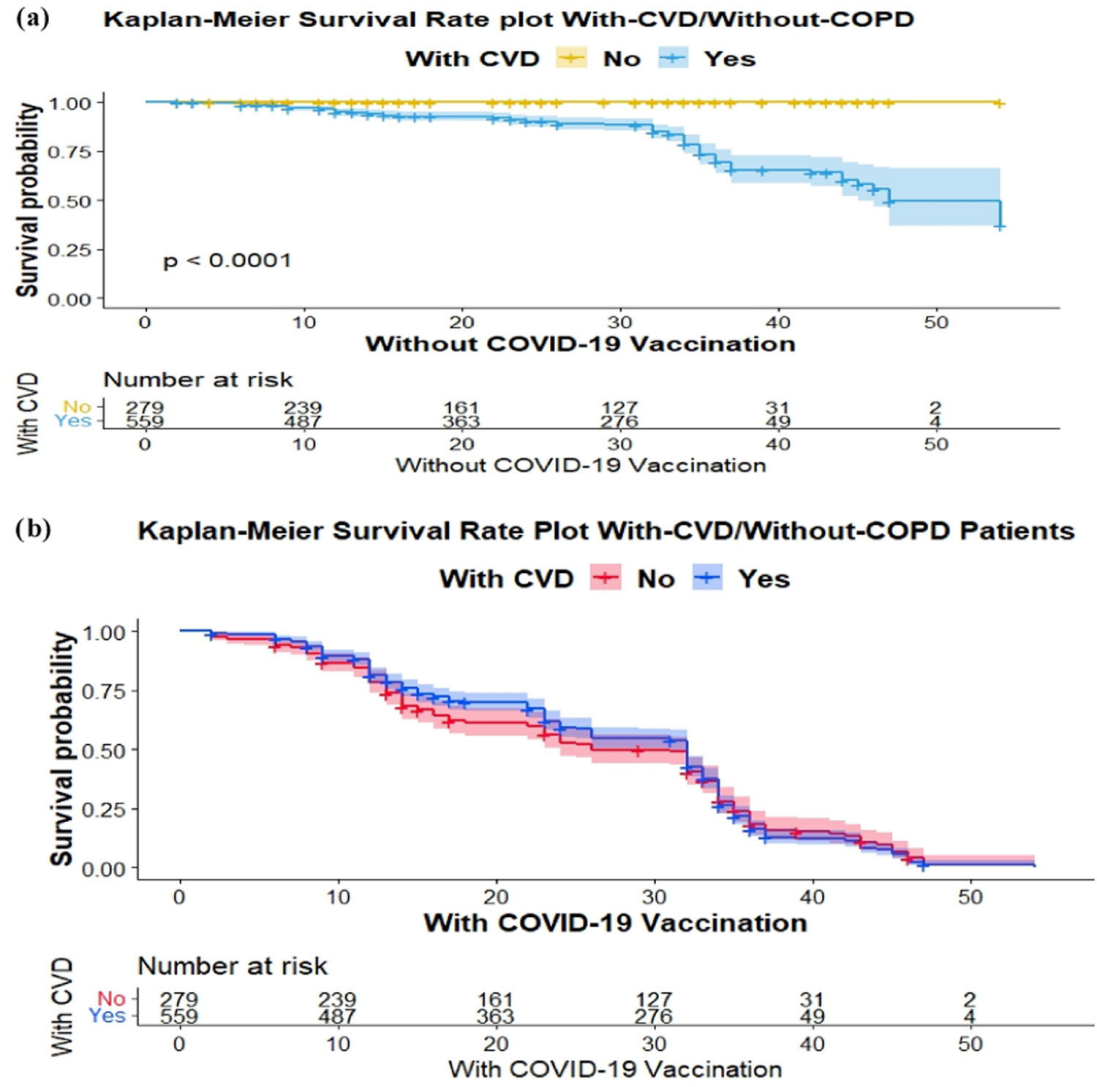
Survival analysis. (a) Without COVID‐19 vaccination versus with‐CVD/without‐COPD Kaplan–Meier curve, time‐to‐death, and survival evaluated the survival function. (b) With COVID‐19 vaccination versus patients with‐CVD/without‐COPD Kaplan–Meier curve, time‐to‐death, and survival evaluated of COVID‐19‐vaccinated patients. COPD, chronic obstructive pulmonary disease; CVD, cardiovascular disease.

**Figure 2:**
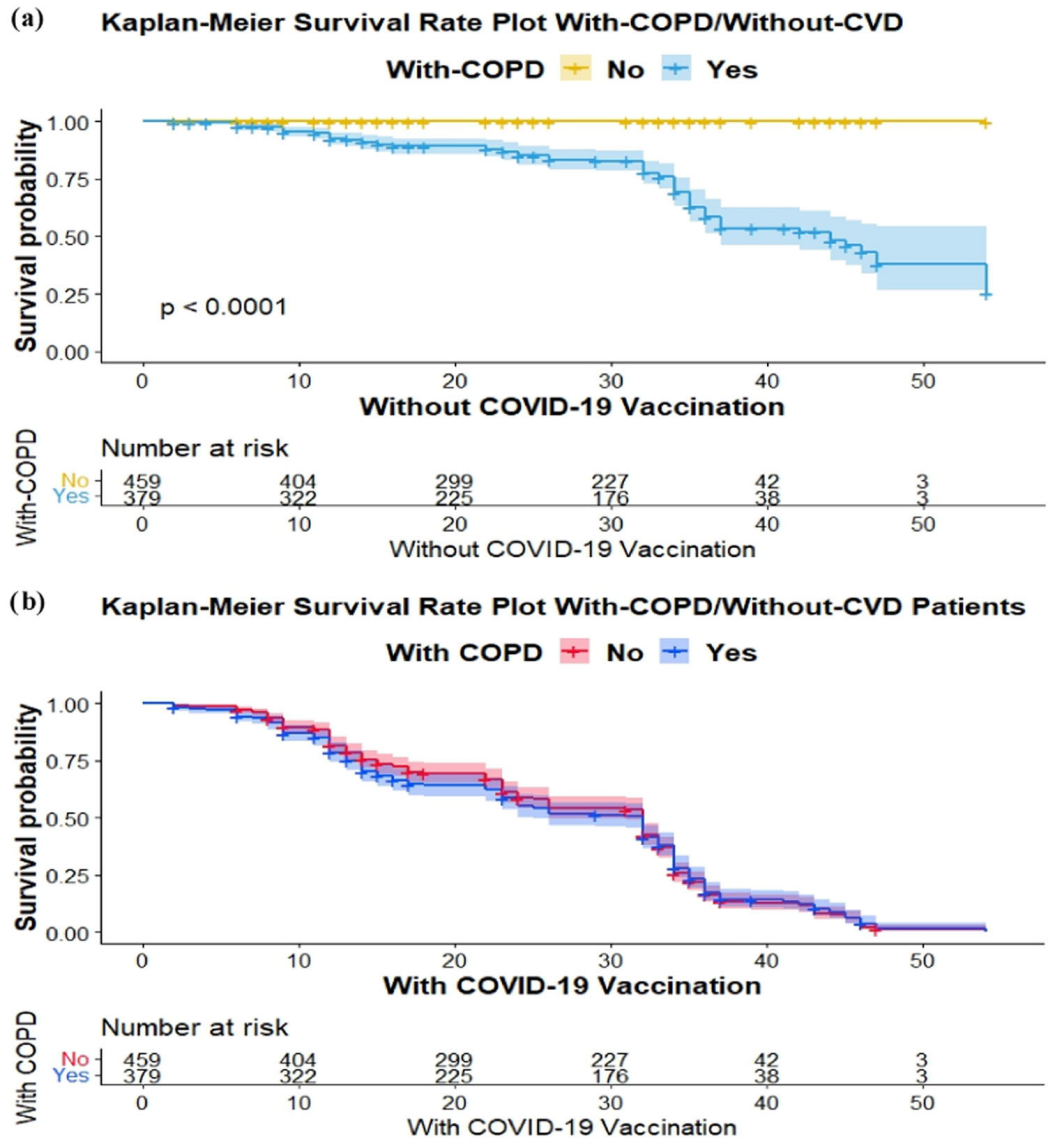
Survival analysis. (a) Without COVID‐19 vaccination versus with‐COPD/without‐CVD Kaplan–Meier curve, time‐to‐death, and survival evaluated the survival function. (b) With COVID‐19 vaccination versus patients with‐COPD/without‐CVD Kaplan–Meier curve, time‐to‐death, and survival evaluated of COVID‐19‐vaccinated patients. COPD, chronic obstructive pulmonary disease; CVD, cardiovascular disease.

Kaplan–Meier curves depicted the results pattern of the survival graph proposed for COPD with CVD patient vaccination history status as shown in Figures 1 and 2. The survival graphs in Figure 1a,b provide an overview of the typical progression of patient outcomes. The results confirm that patients with‐CVD had a significantly higher chance of survival as compare to without‐COPD patients. Kaplan–Meier curves for patients with‐CVD and without‐COPD that received the COVID‐19 vaccine showed a higher probability of survival over time compared with those who did not receive the COVID‐19 vaccine.

The survival graphs in Figure 2a,b provide an overview of the typical progression of patient outcomes. The results confirm that patients with‐COPD had a significantly higher chance of survival as compare to without‐CVD patients. Kaplan–Meier curves for patients with‐COPD and without‐CVD that received the COVID‐19 vaccine showed a higher probability of survival over time compared with those who did not receive the vaccine. This could imply that COVID‐19 vaccination is particularly beneficial for patients with‐COPD/without‐CVD.

When one unit increases in the age, area, DM, HTN, obesity, event, vaccination of COVID‐19, without vaccination of COVID‐19, without‐CVD, and without‐COPD for smoking history in unstandardized coefficients when all other variables remain constant, it increases 1.03, 0.02, 0.90, 0.92, 0.05, 0.47, 0.25, 0.98, 0.16, and 0.25, units. On the other hand, when one unit increases in gender, socioeconomic status, and duration of inhaler, dyslipidemia, and community‐acquired pneumonia, then for smoking history in unstandardized coefficients when all other variables remain constant, it decreases by 0.43, 0.15, 0.01, 0.50, and 0.15 units, as shown in Table 2.

**Table 2:** Coefficients table of multiple regression analysis of COPD and CVD patients.

Factors^a^^,^ b	Coefficients (SE)	OR (95% CI)	*p* value
**(Constant)**	0.65 (0.19)	—	0.001
Sex	−0.43 (0.09)	−0.11 (−0.62–0.23)	0.000
**Age**	1.03 (0.079)	0.39 (0.18–1.19)	0.000
Area	0.02 (0.02)	0.02 (−0.02–0.08)	0.283
Socioeconomic status	−0.15 (0.08)	−0.05 (−0.31–0.00)	0.057
**Diabetes mellitus**	0.90 (0.08)	0.33 (0.24–1.06)	0.000
**Hypertension**	0.92 (0.08)	0.35 (0.05–1.09)	0.000
**Obesity**	0.05 (0.08)	0.02 (−0.10–0.21)	0.475
Duration of inhaler	−0.01 (0.02)	−0.01 (−0.00–0.03)	0.533
Dyslipidemia	−0.50 (0.07)	−0.21 (−0.035–0.35)	0.000
Community‐acquired pneumonia	−0.15 (0.11)	−0.04 (−0.03–0.07)	0.185
**With COVID‐19 vaccination**	0.25 (0.11)	0.07 (0.03–0.47)	0.022
**Without COVID‐19 vaccination**	0.98 (0.09)	0.29 (0.08–1.47)	0.004
Event	0.47 (0.13)	0.22 (0.19–0.54)	0.001
Without CVD	0.16 (0.07)	0.06 (0.01–0.31)	0.035
Without COPD	0.25 (0.03)	0.07 (0.02–0.14)	0.055

Abbreviations: CI, confidence interval; COPD, chronic obstructive pulmonary disease; CVD, cardiovascular disease; OR, odds ratio.

^a^ Dependent variable: Smoking.

^b^ Predictors: (Constant), gender, age, area, socioeconomic status, diabetes mellitus, hypertension, obesity, duration of inhaler, dyslipidemia, community‐acquired pneumonia, with vaccination, without vaccination, event, without CVD, without COPD.

**Figure 3:**
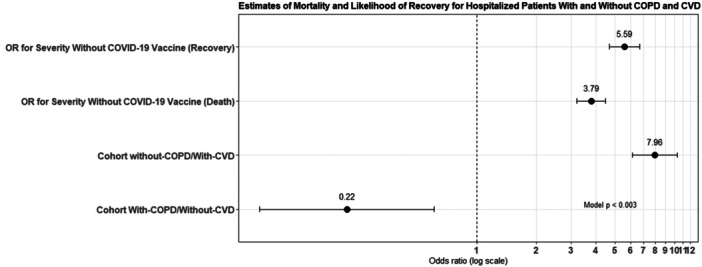
Estimation of mortality and likelihood of recovery for hospitalized patients with and without COPD and CVD. COPD, chronic obstructive pulmonary disease; CVD, cardiovascular disease.

Patients for severity without COVID‐19 vaccine had a 7.958 times higher mortality rate than those with it (95% CI, 6.12–10.33). Patients had a lower chance of recovery at 0.22 (95% CI, 0.08–0.60). When hospitalized patients were assessed, those for severity with COVID‐19 vaccine had a similarly higher mortality rate. The estimates of relative risk (RR) with‐COPD/without‐CVD were at 5.59 (95% CI, 4.68–6.67), and the estimates of RR without‐COPD/with‐CVD observed at 3.79 (95% CI, 3.20–4.48), which denoted greater than the estimates of RR in with‐COPD/without‐CVD patients as shown in Table 3 and Figure 3. The Wilcoxon signed‐rank test clearly highlighted that the null hypothesis is totally rejected at *p* < 0.05, the median of differences between with or without COPD and CVD.

**Table 3:** Estimates of mortality and likelihood of recovery for hospitalized patients with and without COPD and CVD disease were comparable.

Matched cohort with‐COPD/without‐CVD (*n* = 379) without‐COPD/with‐CVD (*n* = 559)	RR (95% CI)	*p* value
Odd ratio for severity without COVID‐19 vaccine (death)	7.95 (6.12–10.33)	0.003
Odd ratio for severity without COVID‐19 vaccine (recovery)	0.22 (0.08–0.60)	
For cohort with‐COPD/without‐CVD	5.59 (4.68–6.67)	
For cohort without‐COPD/with‐CVD	3.79 (3.20–4.48)	

Abbreviations: CI, confidence interval; COPD, chronic obstructive pulmonary disease; CVD, cardiovascular disease; RR, relative risk.

## 4 Discussion

In this research, we performed a comprehensive investigation into the complex relationship between two significant threats to human health: COPD and CVD. Still these problems create a complex network of difficulties for millions of people around the world; we assessed to uncover the hidden relationships that are often ignored. The study centered on a cohort of patients who explored the complex interplay between smoking, COPD, and CVD, revealing novel connections that challenge established beliefs. Throughout the process of our investigation, unexpected protective measures emerged, which affected our comprehension of these health dynamics.

The findings of this retrospective study provide insights into the intricate interplay among COPD, CVD, and COVID‐19. Our finding explores the patients with COPD and CVD may have a higher likelihood of being targeted for the COVID‐19 vaccine, potentially influencing vaccination strategies. Factors like age, DM, HTN, obesity, event, severity with vaccination of COVID‐19, severity without vaccination of COVID‐19, without‐CVD, and without‐COPD for smoking history were positively associated. Smoking history was negatively linked with COPD, CVD, and COVID‐19 for gender, socioeconomic status, inhaler duration, dyslipidemia, and community‐acquired pneumonia.

The Kaplan–Meier survival graphs are utilized to illustrate the impact of CVD, COPD, COVID‐19, and smoking history on the overall prognosis of patients. The results indicated that individuals diagnosed with CVD exhibited a markedly elevated likelihood of survival. The data presented in Figure 1b demonstrates that individuals who were diagnosed with CVD and did not have COPD but received the COVID‐19 vaccine exhibited a greater likelihood of survival compared with those who did not receive the vaccine. Conversely, as depicted in Figure 2b, individuals diagnosed with COPD exhibited a notably higher probability of survival compared with individuals without CVD. Furthermore, the management of the COVID‐19 vaccine has been shown to enhance long‐term survival rates among individuals with COPD as well as CVD. The findings of this study provide guidance for individuals, both with and without COPD, who have a vaccination history and could potentially derive advantages from being, administered the COVID‐19 vaccine. Nevertheless, individuals diagnosed with both COPD and COVID‐19 who were administered corticosteroids exhibited a reduced likelihood of mortality compared with those who did not receive such treatment.

It also explores that patients diagnosed with both COPD and COVID‐19 who were administered corticosteroids exhibited a reduced likelihood of mortality compared with those who did not receive this treatment. The adjusted hazard ratio (HR) was calculated to be 0.61 (95% CI, 0.38–0.99) [22]. According to a population‐based study conducted in Scotland, individuals diagnosed with both COPD and CVD exhibited a considerably elevated risk of hospitalization and mortality due to COVID‐19 when compared with those without these comorbidities. The study found an HR of 2.99 (95% CI, 2.05–4.37) for hospitalization and 3.70 (95% CI, 1.82–7.52) for death in this particular population [23].

The study is subject to various limitations, such as its retrospective nature and dependence on medical records. The study did not take into account the comorbidities and disease severity of the patients. The findings of this study suggest the presence of complex interrelationships among COPD, CVD, COVID‐19, and vaccination. Further investigation is required to comprehend these associations. The vaccine may be less effective in COPD and CVD patients due to immune dysfunction or other factors.

It is to be observed that the mortality rate is high due to severe COVID‐19 in COPD and CVD patients. The healthcare provider should evaluate the benefits and risks of COVID‐19 vaccination in COPD with CVD patients. In susceptible populations where COPD and CVD patients exist, public health policymakers should be vaccinated. Both health and demographic factors may stop the development in patients with COPD and CVD. Risk factors like HTN, DM, and obesity may reduce or alleviate the burden of COPD, CVD, and COVID‐19 and promote healthy lifestyle choices. Additionally, efforts to promote vaccination against COVID‐19 in these vulnerable populations should be a priority to help diminish the burden of the disease [16, 24, 25, 26, 27].

Patients with COPD who also have HIV will have a hard time due to residual confounding. The absence of data regarding HIV/AIDS in our study may have had an impact on our estimates, as it would have been advantageous to have patients with COPD and no comorbidities for the purpose of eliminating other potential factors. Individuals who were not present on a specific date were excluded from the survival analyses. A statistical analysis was conducted to assess the dispersion of potential confounding variables within the sample utilized for each time‐to‐event analysis. The findings of this study suggest the presence of complex interrelationships among COPD, CVD, COVID‐19, and vaccination. There may also be gaps in the information we have regarding the two most important outcomes (survival and mortality). The individuals who did not exhibit any outcome on the final day of follow‐up were the subject of investigation in terms of how the survival analysis method addressed these cases involving left against medical advice. This may involve a multidisciplinary approach that includes respiratory and cardiovascular specialists to optimize outcomes and improve overall health.

## 5 Conclusion

The research highlights the significance of COVID‐19 vaccination for COPD and CVD patients, who may be at higher risk of disease complications. The findings also suggest that factors like age, DM, HTN, obesity, and vaccination status predict patient outcomes. However, the study's retrospective design and reliance on medical records have drawbacks. To confirm these findings and investigate the mechanisms of the observed relationships, more investigation is required. The study highlights the importance of targeted public health policies and clinical management strategies to reduce the burden of COPD, CVD, and COVID‐19 on vulnerable populations.

## Author Contributions

**Muhammad Muneeb Hassan:** conceptualization, data curation, formal analysis, funding acquisition, investigation, methodology, project administration, resources, software, supervision, validation, visualization, writing–original draft, writing–review and editing. **Sheik Muhammad Sikandar:** Contributes to manuscript writing, editing, visually engagement, with standard and final approval of the version to be published. **Farrukh Jamal:** formal analysis, investigation, writing–original draft, writing–review and editing. **Muhammad Ameeq:** data curation, investigation, methodology, resources, software, supervision, validation, visualization, writing–original draft, writing–review and editing. **Alpha Kargbo:** conceptualization, data curation, formal analysis, funding acquisition, investigation, methodology, project administration, resources, software, supervision, validation, visualization, writing–original draft, writing–review and editing.

## Ethics Statement

There were no objections or ethical concerns raised by the Ethical Review Committee of District Hospital Muzaffargarh, Punjab, Pakistan. On April 7, 2023, No. 1107‐11/DHQ the Statistical Officer in Charge of the Ethical Committee at DHQ Hospital in Muzaffargarh, Punjab, Pakistan approved the plan for the study to be conducted in accordance with the Declaration of Helsinki.

## Conflicts of Interest

The authors declare no conflicts of interest.

## Data Availability

The data that supports the findings of this study are available on request from the first author, Muhammad Muneeb Hassan. The data are not publicly available due to restrictions on their containing information that could compromise the privacy of research participants. The corresponding author can provide access to the data, models, and code that support the findings of this study.
